# Utility of Hybrid Transferrin Binding Protein Antigens for Protection Against Pathogenic Neisseria Species

**DOI:** 10.3389/fimmu.2019.00247

**Published:** 2019-02-19

**Authors:** Jamie E. Fegan, Charles Calmettes, Epshita A. Islam, Sang Kyun Ahn, Somshukla Chaudhuri, Rong-hua Yu, Scott D. Gray-Owen, Trevor F. Moraes, Anthony B. Schryvers

**Affiliations:** ^1^Department of Microbiology, Immunology and Infectious Diseases, University of Calgary, Calgary, AB, Canada; ^2^Department of Molecular Genetics, University of Toronto, Toronto, ON, Canada; ^3^Department of Biochemistry, University of Toronto, Toronto, ON, Canada

**Keywords:** protein engineering, outer membrane protein, lipoprotein, hybrid antigen, scaffold, epitope

## Abstract

The surface transferrin receptor proteins from *Neisseria gonorrhoeae* have been recognized as ideal vaccine targets due to their critical role in survival in the human male genitourinary tract. Recombinant forms of the surface lipoprotein component of the receptor, transferrin binding protein B (TbpB), can be readily produced at high levels in the *Escherichia coli* cytoplasm and is suitable for commercial vaccine production. In contrast, the integral outer membrane protein, transferrin binding protein A (TbpA), is produced at relatively low levels in the outer membrane and requires detergents for solubilization and stabilization, processes not favorable for commercial applications. Capitalizing on the core β-barrel structural feature common to the lipoprotein and integral outer membrane protein we engineered the lipoprotein as a scaffold for displaying conserved surface epitopes from TbpA. A stable version of the C-terminal domain of TbpB was prepared by replacing four larger exposed variable loops with short linking peptide regions. Four surface regions from the plug and barrel domains of *Neisseria* TbpA were transplanted onto this TbpB C-lobe scaffold, generating stable hybrid antigens. Antisera generated in mice and rabbits against the hybrid antigens recognized TbpA at the surface of *Neisseria meningitidis* and inhibited transferrin-dependent growth at levels comparable or better than antisera directed against the native TbpA protein. Two of the engineered hybrid antigens each elicited a TbpA-specific bactericidal antibody response comparable to that induced by TbpA. A hybrid antigen generated using a foreign scaffold (TbpB from the pig pathogen *Haemophilus parasuis)* displaying neisserial TbpA loop 10 was evaluated in a model of lower genital tract colonization by *N. gonorrhoeae* and a model of invasive infection by *N. meningitidis*. The loop 10 hybrid antigen was as effective as full length TbpA in eliminating *N. gonorrhoeae* from the lower genital tract of female mice and was protective against the low dose invasive infection by *N. meningitidis*. These results demonstrate that TbpB or its derivatives can serve as an effective scaffold for displaying surface epitopes of integral outer membrane antigens and these antigens can elicit protection against bacterial challenge.

## Introduction

*Neisseria gonorrhoeae*, the causative agent of the sexually transmitted infection gonorrhea, relies on acquiring iron from the host iron-binding glycoproteins, transferrin (Tf) or lactoferrin (Lf), for survival in the human genitourinary tract (1, 2). This process is mediated by surface receptor proteins initially discovered in the related human pathogen *Neisseria meningitidis* (3, 4) and are presumed to be essential for survival of *N. meningitidis* both in the human upper respiratory tract during asymptomatic colonization and during invasive infection. The bacterial Tf and Lf receptor systems are each composed of an anchored lipoprotein, Tf or Lf binding protein B (TbpB, LbpB) that extends away from the bacterial surface to bind iron-loaded Tf or Lf, and the integral membrane proteins Tf or Lf binding protein A (TbpA, LbpA) that transfer iron across the outer membrane. These receptors are each exquisitely specific for human Tf and Lf (5), extending detectable binding activity only to apes but not monkeys (6). The strict specificity of TbpA for Tf was shown to be the result of mutations in sites on Tf critical for TbpA binding in response to selective pressure by the presence of the bacterial receptor proteins (7), indicating that the specificity co-evolved over 40 million years of primate divergence. It is noteworthy to recognize that the TbpA-Tf interaction is present in pathogens of multiple important food production animals, including poultry, swine, and cattle, (8, 9), suggesting that the host specificity has evolved over a period of more than 300 million years, when the Synapsids (mammalian lineage) split from Sauropsids (bird, reptile lineage).

Even prior to the experimental demonstration of the importance of the Tf receptor proteins for bacterial survival and disease causation, their presumed importance made them attractive candidates for vaccine development. The receptor complex comprised of TbpA and TbpB were shown to protect against meningococcal infection in mice (10) and shown to induce protective antibodies against *N. meningitidis* in laboratory animals (11). Although the integral outer membrane TbpA (previously denoted Tbp1) was, with notable exception (12), the essential component for *in vitro* growth (13, 14), the surface lipoprotein, TbpB (previously denoted Tbp2) became the focus for meningococcal vaccine development due to the protective immune response from purified native receptor proteins being predominantly associated with TbpB (15). The encouraging results from ongoing experiments with laboratory animals (16) led to implementing a Phase I trial in humans that was not sufficiently encouraging (17) to continue TbpB-focused meningococcal vaccine development efforts.

In order to test the potential efficacy of full-length TbpB and TbpA as vaccine antigens for protection against gonococcal infection, the intact proteins were coupled to cholera toxin B subunit (Ctb) and used for intranasal immunization of mice (18). The Ctb conjugates induced serum and vaginal antibodies and although the anti-TbpB titres were higher, the anti-TbpA antibodies were more cross-reactive. In a follow up study, regions of TbpB (N-lobe) and TbpA (loop 2) were genetically fused to the cholera A2 toxin subunit and these preparations were able to induce serum and vaginal antibodies that conferred bactericidal activity and inhibited growth dependent upon exogenous Tf (19). These results indicated that individual loop regions of TbpA were capable of inducing functional antibodies targeting intact TbpA.

Given its superior efficacy in mouse and rabbit-based studies, we hypothesized that the unexpected performance of recombinant *Neisseria* TbpB in the human Phase I trials was due to human Tf blocking important epitopes on the immunizing antigen, a limitation which would not have been present when immunizing laboratory animals due to the specificity of TbpB binding for human Tf. In an attempt to evaluate this postulate in the natural host of a Tbp-expressing pathogen, we exploited an established pig infection model. To this end, a single residue mutant of TbpB from the porcine pathogen *Haemophilus parasuis* that was defective in its binding to porcine Tf was compared to the native TbpB and a commercial vaccine product in an immunization and challenge experiment in pigs (20). The superior protection against lethal challenge provided by the non-binding *H. parasuis* TbpB protein compared to either native (wild type) TbpB or the commercial *H. parasuis* vaccine in this experiment suggests that it is worth reconsidering TbpB-based vaccines in humans. The demonstration that a Tf binding-defective *H. parasuis* TbpB is capable of inducing a cross-protective immune response against a heterologous strain expressing a TbpB variant from the same phylogenetic cluster (21) suggests that vaccine compositions with a limited number of recombinant, engineered TbpBs should be capable of inducing a comprehensive, cross-protective immune response. Taken together with the ability to produce high levels of functional and stable recombinant TbpB in the *Escherichia coli* cytoplasm, the prospects for developing TbpB-based vaccines is enticing.

Recombinant TbpA extracted and purified from the bacterial outer membrane has been shown to be effective at preventing meningococcal sepsis in a mouse infection model (22) and inducing *N. gonorrhoeae*-specific serum and vaginal antibodies in mice (18). However, there are many potential barriers to commercial production of recombinant TbpA as a vaccine antigen. Efficient production of functional, accurately folded TbpA requires insertion into the outer membrane, a process that limits production yield. The need for detergent to extract and purify TbpA and either detergents or other amphipathic reagents for maintaining stability and solubility of purified TbpA are substantial barriers for commercial development. Thus, serious consideration of TbpA as a vaccine target may require novel approaches for generating antigens capable of inducing a protective anti-TbpA response.

Protein crystallography of TbpBs and TbpB-Tf complexes (23–25) have revealed TbpB as a bi-lobed protein with individual N-terminal and C-terminal lobes each comprised of two adjacent anti-parallel β-barrel like structures with relatively unstructured loops linking the β-strands. The TbpBs thus share common features with the β-barrels of integral outer membrane proteins such as TbpA (26), however the barrel surfaces are hydrophilic such that soluble, stable recombinant proteins can be produced. We initiated a study to determine whether TbpB or its lobes could function as a scaffold to display loops from TbpA that would be capable of eliciting functional antibody against TbpA, resulting in a protective immune response. Herein, we demonstrate that these novel antigens are able to protect against *N. gonorrhoeae* in a mouse model of mucosal colonization and against acute infection by *N. meningitidis* in a mouse model of sepsis.

## Materials and Methods

### Antigen Engineering

**Figure 1:**
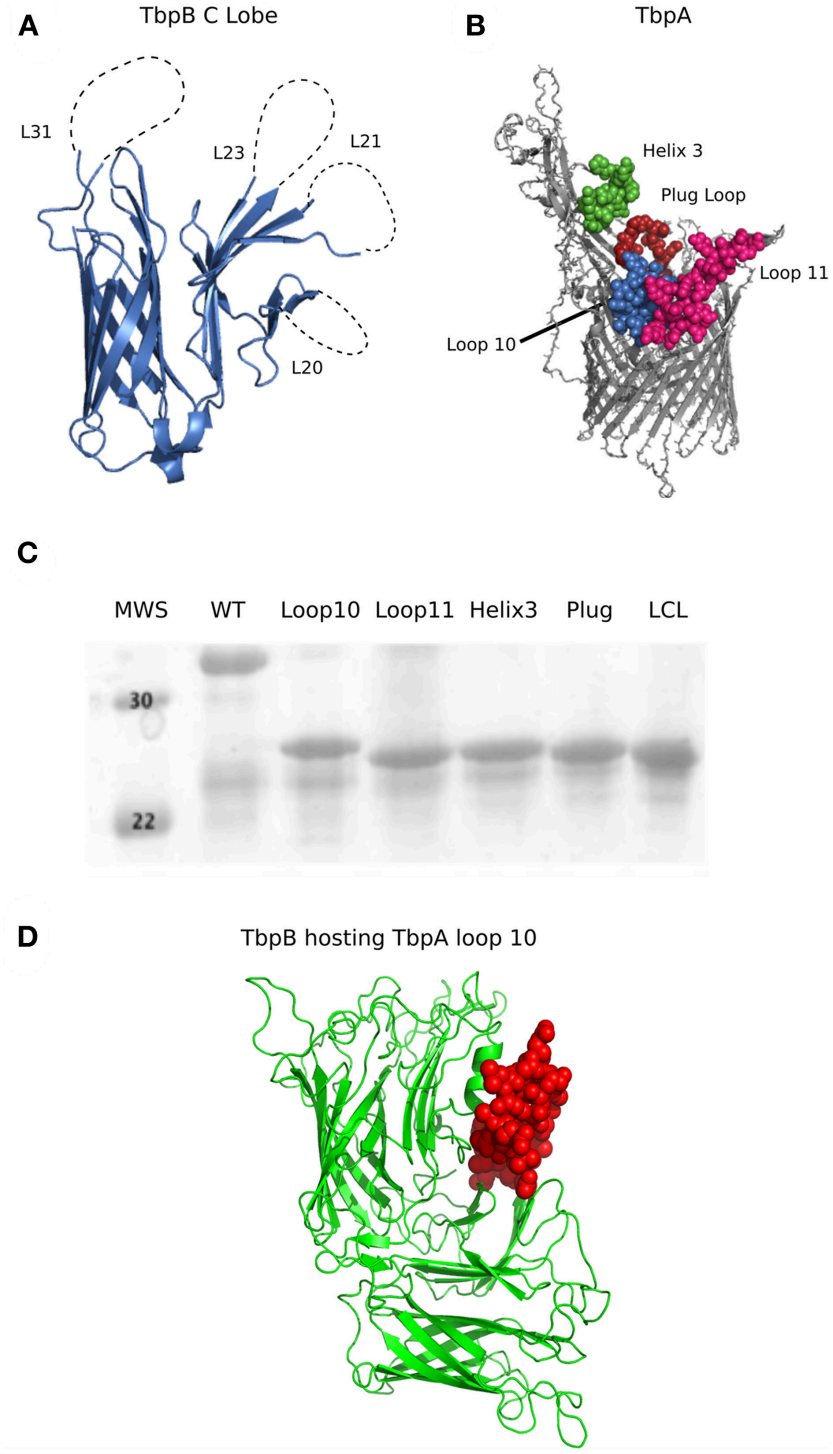
**(A)** Cartoon diagram of the solved crystal structures of *N. meningitidis* TbpB C Lobe (PDB 3VE2) from strain M982. Loop 20 (26 amino acids; 413–439), loop 21 (30 amino acids; 444–474), loop 23 (21 amino acids; 499–520) and loop 31(21 amino acids; 657–676) are illustrated as dotted lines. **(B)** Surface regions of TbpA targeted for display on the hybrid antigens. Cartoon diagram of *N. meningitidis* M982 TbpA modeled with Phyre2 (27) using PDB 3V89 as a template, with the loop structures of interest shown in green (loop 3 helix), red (plug domain), pink (loop 11) and blue (loop 10). Structures on the apical side are surface facing while beta strands are within the outer membrane. **(C)** SDS-PAGE of the proteins produced from the genes encoding the wildtype C lobe, the LCL, and the four TbpA-LCL hybrid antigens that were cloned and purified. **(D)** Cartoon diagram of a Phyre2 model of a *Neisseria* TbpB (green) hosting TbpA loop 10 (red) as an example of a novel hybrid antigen displaying an exogenous loop.

**Figure 2:**
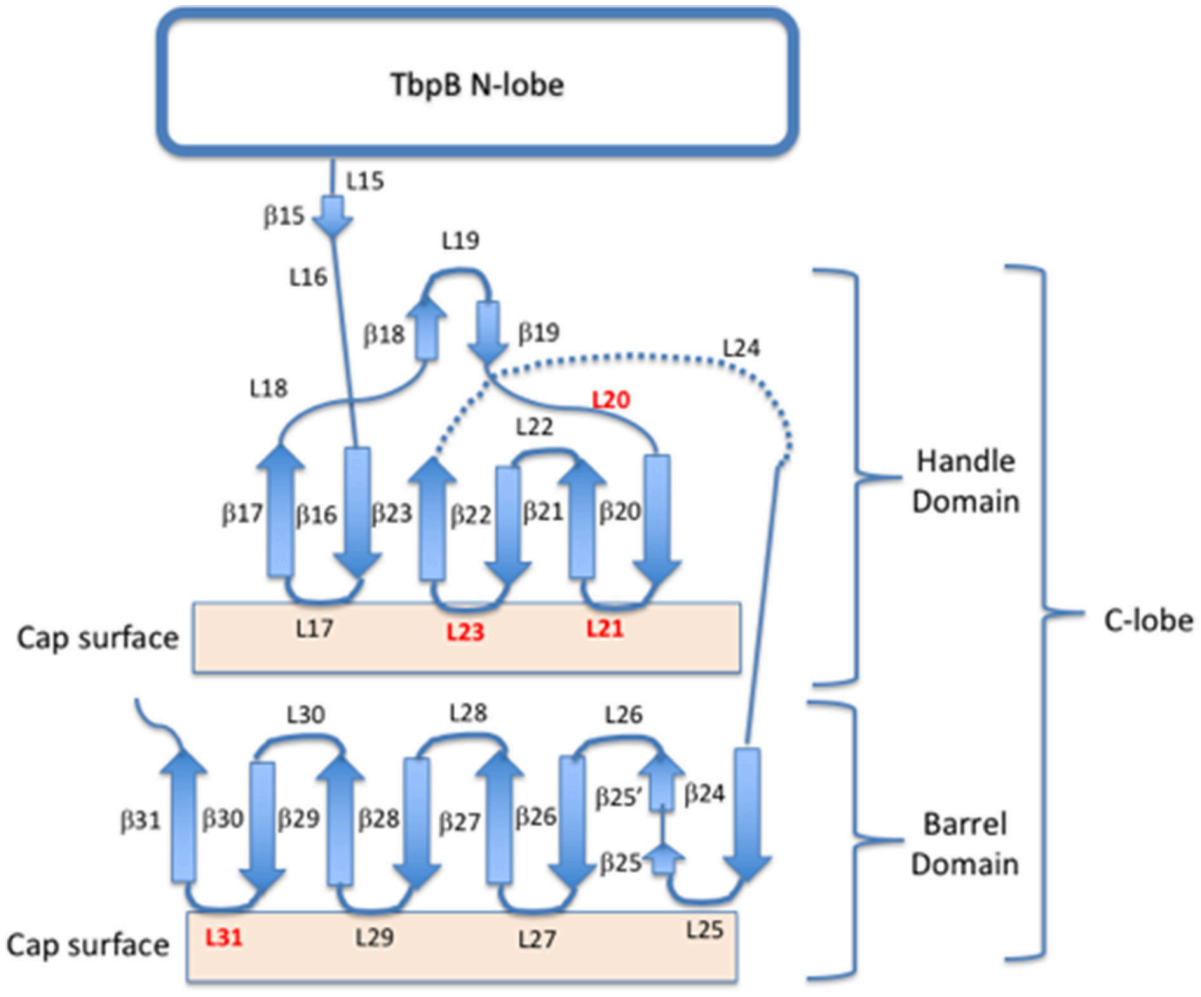
Cartoon schematic of the insertion sites for exogenous loops on the C lobe of TbpB. Four insertion sites (loops 20, 21, 23, and 31) are shown in red, while the remaining loops and beta-sheets are annotated for both the barrel and handle domains of the C-lobe.

The crystal structures of TbpA from *N. meningitidis* strain MC58 (PDB 3V8X) and TbpB from *N. meningitidis* strain M982 (PDB 3VE2) were used as a basis for antigen design. A computational model of TbpA from *N. meningitidis* strain M982 was generated using the Phyre2 server (27) with PDB 3V8X as a template. The C-lobe of the *N. meningitidis* strain M982 TbpB (TbpB^377−689^) was selected as a scaffold (Figure 1A). The β-strands flanking the four loops on the TbpB C-lobe that were not resolved in the crystal structure (loop 20 ^413−439^; loop 21^444−474^; loop 23^499−520^; loop 31^657−676^) were selected as the sites for insertion of foreign epitopes. Loops 21, 23, and 31 are standard β-barrel loops connecting two immediately adjacent anti-parallel β-strands whereas loop 20 connects two β-strands that are quite separate as illustrated in Figure 2 (24). The original loops were of a reasonable size (26, 30, 21, and 19 amino acids, respectively) suggesting that these locations would be amenable to hosting foreign loops of a similar size. These loops were replaced by short sections from the equivalent loops of the *Actinobacillus pleuropneumoniae* TbpB, generating the “loopless” C-lobe (LCL). The LCL was used as a scaffold to display four surface exposed regions from *N. meningitidis* strain M982 TbpA; loop 3 helix (TbpA^350−363^), loop 10 (TbpA^815−843^), loop 11 (TbpA ^875−901^) and the plug loop (TbpA^123−139^). These loops were inserted into the sites of TbpB loops 20, 21, 23, and 31, respectively. Notably, loop 20 was selected for hosting the loop 3 helix region of TbpA, which is not a typical individual loop, since the anchor points were far enough apart to accommodate this region. The regions encoding foreign epitopes were introduced into the gene encoding the LCL by SOE (splicing by overlap extension)-PCR (28). The primers used for preparation of the LCL and the individual loop hybrids are listed in Table 1. We opted to use a scaffold lacking the N-lobe to potentially enhance the immune response against the individual loops.

**Table 1:** Oligonucleotide Primers.

**No**.	**Name**	**Protein sequence**	**Sequence**
4012	C-lobefor	GSSSENK	GCGC**GGATCC**TCGTCTGAAAACAGTAAGCTG
3181	C-loberev	KRQQPVQ^*^	ATGCCAGT**AAGCTT**TTATTGCACAGGCTGTTGGCGTTTC
4046	LCLlp1rev	VDGGIMIDLAG	TCCGGCCAGATCAATCATAATGCCGTCGAC
4045	LCLlp1for	ILDLAGTEFTR	ATTGATCTGGCCGGAACAGAATTTACCCGC
4048	LCLlp2rev	RKFEHTTINGK	TTTGCCGTTAATCGTGTGTTCAAATTTGCG
4047	LCLlp2for	TINGKTYEVE	ACGATTAACGGCAAAACCTATGAAGTCGAA
4050	LCLlp3rev	GMLTRKGKQV	AACCTGTTTGCCTTTGCGCGTCAACATTCC
4049	LCLlp3for	KGKQVEQSMF	AAAGGCAAACAGGTTGAACAAAGTATGTTC
4052	LCLlp4rev	GGWFAYHKSDNGS	GCTGCCGTTATCGCTTTTATGATAGGCAAACCATCCGCC
4151	LCLlp4for	YHKSDNGSATVVF	TATCATAAAAGCGATAACGGCAGCGCGACCGTGGTATTC
4157	LCLlp1-helixrev	IPLLPATKAVFDENRK	ATTTGCGGTTCTCATCAAAGACGGCTTTTGTGGCTGGGAGGAGTGGAAT
4156	LCLlp1-helixfor	AVFDENRKYGPEFTRKFE	GCCGTCTTTGATGAGAACCGCAAATACGGCCCGGAATTTACCCGCAAATTTGAA
4159	LCLlp2-lp10rev	TRKFEHTAKEITELLGSRALLNGNSRN	ATTGCGGCTGTTGCCGTTGAGCAAAGCCCGGCTGCCCAACAACTCTGTGATTTCCTTGGCCGTGTGTTCAAATTTGCGGGT
4158	LCLlp2-lp10for	SRALLNGNSRNTKATARRTRTYEVEVC	AGCCGGGCTTTGCTCAACGGCAACAGCCGCAATACAAAAGCCACCGCGCGCCGTACCCGCACCTATGAAGTCGAAGTCTGC
4161	LCLlp3-Lp11rev	YGMLTRKVTWENVRQTAGGAVNQHK	TTGTGTTGGTTGACTGCGCCGCCGGCAGTTTGCCGCACATTTTCCCAAGTAACTTTGCGCGTCAACATTCCGTA
4160	LCLlp3-Lp11for	AGGAVNQHKNVGVYNRYAVEQSMFL	GCCGGCGGCGCAGTCAACCAACACAAAAATGTCGGCGTTTACAACCGATATGCCGTTGAACAAAGTATGTTCCTC
4163	LCLlp4-pluglprev	LGGWFAYTAQAALGGTRT	CGTCCTCGTCCCGCCCAATGCCGCCTGCGCGGTATAGGCAAACCATCCGCCCAA
4162	LCLlp4-pluglpfor	AALGGTRTAGSSATVV-FG	GCGGCATTGGGCGGGACGAGGACGGCGGGCAGCAGCGCGACCGTGGTATTCGGT
4755	HpTbpBfor	GSGVSKE	TGAACA**GGATCC**GGCGTGTCTAAAGAAG
4756	HpTbpBrev	GAKQQVKK	TGTTCA**ACTAGT**TTATTTTTTTACTTGTTGTTTTGCACC
4772	RvsPos4uplp10	LGGQFRYQKAKE	CCTTGGCTTTTTGATAACGGAATTGTCCGCCAA
4773	FwdPos4uplp10	GQFRYQKAKEITELLG	GACAATTCCGTTATCAAAAAGCCAAGGAAATCACAGAGTTGTTGGG
4774	Revlp10Pos4dn	ARRTRPVGVGAVFGAKQQ	CTTGTTGTTTTGCACCAAAGACAGCACCTACACCTACAGGGCGGGTACGGCGCG
	TbpBKO_UpF		ATGACGCGATTAGAGTTTCA
	TbpBKO_UpR	MNNPLVI (TbpB)	GGATATCAGCTGGATGGCAATCACCAATGGATTGTTCATA
	TbpBKO_DownF		TAATTGGTTGTAACACTGGCAGCACGGCTGCCGAACAATC
	TbpBKO_DownR	YSIRGMD (TbpA)	CCATACCGCGTATCGAGTAG
4600	Kan_F		TTGCCATCCAGCTGATATCC
4601	Kan_R		GCCAGTGTTACAACCAATTA

* *Underlined residues are sequences of the inserted loops*.

To produce a hybrid antigen where the Neisseria TbpA loop 10 was hosted on a “foreign” porcine pathogen TbpB for better evaluation of protection mediated by TbpA alone, the mutant TbpB structure (PDB 4O4U) from *Haemophilus parasuis* serovar 7 was used for antigen design (Figure 1D). Neisseria loop 10 TbpA^810−840^ was inserted into TbpB loop 31 (TbpB ^518−522^) of a non binding mutant of *H. parasuis* TbpB (TbpB^20−537^ Y167A) by SOE-PCR and has been denoted as Hp7 + L10. The primers used for preparation of the Hp7 + L10 hybrids are listed in Table 1.

Engineered proteins were produced in *E. coli* strain ER2566 as described previously (20, 29). A recombinant his-tagged form of TbpA was extracted from intact cells with Elugent and purified as described previously (29) except that the Elugent was exchanged with 2% Triton X-100, and upon dilution into buffer used for immunization resulted in a final concentration less than 0.2% Triton X-100.

### Phylogenetic Evaluation

Protein sequences from Neisseria species TbpA and TbpB were collected from the Bacterial Isolate Genome Sequence Database for a total number of 4,176 TbpAs and 1,399 TbpBs. Multiple sequence alignments were done using Clustal Omega v1.2.4 (30, 31). Eight sequences were removed from the TbpA alignment that had substantial sequencing or annotation errors, leaving 4,168 TbpA sequences, and 1,399 TbpB sequences. Phylogenetic trees were built using RaxML v8.2.12 with the WAG evolutionary model and a gamma distribution of rates across sites (32).

Sequences of TbpAs from a panel of *N. meningitidis* and *N. gonorrhoeae* strains were evaluated for loop variability. Clustal Omega was used to produce a multiple sequence alignment (30, 31). The alignment was compared to the solved structure of MC58 TbpA [PBD #3V8X; (26)] to determine loop junctions. Alignments were visualized with Jalview 2 (33).

### Animal Immunizations for *in vitro* Characterization of Serum

Female FvB mice (Charles River, seven weeks old) and New Zealand White rabbits (Reimans Fur Ranches, 3 months old) were immunized with 25 and 50 μg of antigen, respectively. Sub-cutaneous immunizations were performed on days 0, 21 and 42 and were composed of 20% Emulsigen D (MVP adjuvants) in sterile PBS to a final volume of 100 μl (mice) or 500 μl (rabbits) and a final cardiac bleed was done on day 56. Animal immunizations and bleeds were performed in accordance with the University of Calgary Animal Care Committee under protocol AC11-0033

### Strain Construction

A TbpB knockout strain of *N. meningitidis* M982 was constructed by taking advantage of natural transformation. Flanking regions upstream and downstream of the *tbpB* gene were PCR amplified and a kanamycin resistance cassette was inserted between the regions. The PCR amplified DNA was mixed with a cell suspension that was applied to chocolate agar plates and incubated overnight. The cells were then re-suspended and plated onto PC55 chocolate agar (Enriched, Dalynn Biologicals) with 50 μg/ml kanamycin for selection and were confirmed by sequencing. Primers used to prepare the *tbpB* knockout strain are listed in Table 1.

### Whole Cell ELISA

*N. meningitidis* and *N. gonorrhoeae* were grown overnight on chocolate agar and colonies were resuspended in BHI supplemented with 100 μmol/L deferoxamine mesylate salt (“desferal,” Sigma) and grown for 6 h. Cells were resuspended in phosphate buffered saline (PBS) and heat killed at 56°C for 30 min. Cells were resuspended to an OD_600_ 0.4–0.5 in PBS and either 50 μl per well was added to 96-well plates or 20 μl per well was added to 384-well plates and dried overnight. Plates were washed with PBST and then blocked at room temperature with 5% skim milk or 5% bovine serum albumin (BSA) in PBST for 1 h. Sera were added in two-fold dilutions starting at a dilution of 1:500, followed by incubation with a secondary antibody [1:50,000 dilution, Anti-Mouse IgG (H&L) peroxidase antibody (Rockland Inc.) or 1:10,000 dilution, Goat Anti-Mouse IgG (H&L) peroxidase antibody (Jackson ImmunoResearch Laboratories Inc.)], washed and developed (tetra-methyl benzoate, TMB, Sigma). Development was quenched with 1 M H_2_SO_4_ and plates were read at 450 nm.

### Growth Assay

Due to limited volumes of mouse sera, these experiments were performed with rabbit sera. *N. meningitidis* M982ΔtbpB was grown overnight on chocolate plates at 37°C with 5% CO_2_ and then sub-cultured into BHI supplemented with 100 μmol/L desferal and grown for 30 min. 10% v/v heat-killed rabbit sera were added to each culture, grown for 2 h, followed by the addition of hTf and grown for a further 2 h. The viable cell count was determined after dilution and plating on BHI agar grown overnight. LCL sera were initially compared to naïve rabbit sera with no significant differences noted. LCL sera were then used as a control.

### Serum Bactericidal Assay

A modified version of the standard bactericidal assay (34) was used to compare the different sera. *N. meningitidis* strains were grown overnight and re-streaked on chocolate agar with 100 μmol/L desferal and grown for 4 h. Two-fold dilutions of individual heat-killed mouse sera starting at a 1:4 dilution were added to Hank's Buffered Salt Solution (with CaCl_2_ and MgCl_2_, Gibco) and equal volumes diluted bacteria and baby rabbit complement were added (25% v/v each). Bacteria were plated on BHI agar at time zero and after a 60-min incubation at 37°C for enumeration. Bactericidal level was defined as the inverse of the last dilution of sera for which more than 50% of the bacteria were killed compared with the control.

### Lower Genital Tract Infection With *N. gonorrhoeae*

Two cohorts of female 5-week old C57Bl/6 mice (*n* = 9–11 mice per immunization group, per cohort) were immunized three times intra-peritoneally (i.p.) with one of TbpA, Hp7 + L10 or adjuvant (alum; aluminum hydroxide) alone. All immunizations were composed of 25 μg of antigen in sterile PBS formulated with 100 μg aluminum hydroxide (Sigma-Aldrich) in 100 μl total volume, and were administered on day 0, 14, and 28. Mice were treated with three doses of water-soluble 17β-estradiol (0.5 mg per mouse per dose, administered day −2, 0, and 2 relative to the day of infection) to induce susceptibility to gonococcal infection and daily antibiotics (vancomycin, streptomycin and trimethoprim, beginning at day −2) to reduce commensal bacteria in the lower genital tract, as described previously (35, 36). *N. gonorrhoeae* strain MS11 was grown on GC agar supplemented with isovitalex (Fisher Scientific) at 37°C with 5% CO_2_ overnight. Mice were given approximately 10^7^ CFU of *N. gonorrhoeae* resuspended in sterile PBS [with CaCl_2_ and MgCl_2_ (PBS++)] intra-vaginally. Starting on day 1 post infection, vaginal lavages were performed with 10 μl of sterile PBS++ and plated on GC agar supplemented with isovitalex and VCNT inhibitor to determine bacterial burden. Mice that did not have recoverable gonococci on day 1 post infection were removed from analysis, which may be due to perturbations in the vaginal microbiota as has been previously reported (37). Colonies were validated to be gonococci by colony PCR with gonococcal-specific primers. Animal work was performed in accordance with University of Toronto Animal Ethics Review Committee under protocol 20011319.

### Invasive Challenge With *N. meningitidis*

Two cohorts of male C57Bl/6 mice (Charles River, 5 weeks of age) were immunized i.p. three times with one of TbpA or Hp7 + L10 formulated with 100 μg aluminum hydroxide (Sigma-Aldrich), or alum alone (*n* = 4–6 per immunization group, per cohort). Vaccines were formulated with 25 μg of antigen in sterile PBS in a total volume of 100 μl per mouse, and given on day 0, 14, and 28. Sepsis modeling was performed as described previously (38). Briefly, mice were challenged with either 2.5 × 10^7^ CFU (cohort 1) or 7.5 × 10^7^ CFU (cohort 2) of *N. meningitidis* strain M982. *N. meningitidis* was grown overnight on GC agar supplemented with isovitalex (Fisher Scientific) at 37°C with 5% CO_2_. On the day of infection, *N. meningitidis* was resuspended at an OD_600_ of 0.1 in RPMI broth and grown for 4 h with shaking at 37°C. Mice were infected via i.p. injection under isofluorane anesthesia. At the time of infection, mice were also given a separate i.p. injection on the contralateral side containing 8 mg of iron-loaded human transferrin (Sigma-Aldrich) resuspended in sterile PBS. Mice were monitored at 3, 12, 18, 24, 36, and 48 h post infection and tail bleeds were collected at 3, 18, 36, and 48 h post infection or at any time clinical endpoint was reached. Tail bleeds were plated on Difco GC agar (BD Biosciences) supplemented with isovitalex and antibiotic selection (BD BBL VCNT Inhibitor, Fisher Scientific) to determine bacterial burden in the blood. Animal work was performed in accordance with University of Toronto Animal Ethics Review Committee under protocol 20011775.

### Statistical Analyses

Statistical analysis and figures were completed with GraphPad Prism (version 7.0 for Mac OS X; GraphPad, San Diego, CA). Whole-cell ELISA titres were compared with either an ordinary one-way ANOVA with *post-hoc* comparisions performed with Dunnett's multiple comparisons test (**Figures 6A,B**) or a two-way ANOVA with *post-hoc* comparisons performed with Sidak's multiple comparisons test (**Figure 5A**). The ability of sera to block transferrin-based growth was compared with an ordinary one-way ANOVA with *post-hoc* comparisons performed by the Holm-Sidak test (**Figure 5C**). Pre-challenge rabbit serum was compared to LCL immune serum by an unpaired t-test (**Figure 5B**). The duration of gonococcal colonization was evaluated by log rank [Mantel-Cox] test (**Figure 7**).

### Transfer of Methodology

All protocols, methodology and study materials will be made available upon request.

## Results

### Design and Production of Hybrid Antigens

The lobes of the TbpB and LbpB lipoproteins are comprised of two adjacent anti-parallel β-barrel like structures. The loops linking the β-strands can be quite large (> 80 amino acids), particularly the negatively charged regions in the LbpB C-lobe (39), suggesting that the C-lobe can accommodate large loop insertions. Since the loops in the N-lobe region comprise the binding interface with Tf, we considered that targeting the loops in the N-lobe for replacement could interfere with generation of antibodies that could block binding and use of Tf. Therefore, the C-lobe was selected as the preferred scaffold for heterologous epitope display. When designing a scaffold derived from the TbpB of *Neisseria*, we selected as insertion sites the four loops in the C-lobe that were not resolved in the crystal structure of a meningococcal TbpB (25), as these were relatively large and flexible (26, 30, 21, and 19 amino acids, respectively). A preliminary scaffold was prepared by replacing the existing loops on the *N. meningitidis* strain M982 TbpB C-lobe (Figure 1A) with small segments from loop regions of TbpB from *A. pleuropneumoniae* (23).

To test the versatility of the scaffold protein to host exogenous epitopes, we selected four different peptide segments of varying size from surface-exposed regions of the *N. meningitidis* strain M982 TbpA to display. Using the structure of meningococcal TbpA in complex with human Tf as a guide (26), we selected three regions from the extracellular loops extending off the beta-barrel and one from the plug region (Figure 1B) as these regions appeared to be surface exposed and potentially important for TbpA functionality. The two largest inserts consisted of the majority of the sequence of external loops 10 (30 residues) and 11 (27 residues), best representing the effective transplant of substantial segments of external loop regions. The TbpA loop 3 alpha-helix was inserted into the TbpB loop 20 site in which the anchoring β-strands are not adjacent so that the alpha-helical region could be accommodated. Insertion of these individual regions (Figure 1C) or a combination of the four loop insertions (data not shown) did not have a substantial impact on the production or stability of the resulting recombinant proteins.

**Figure 3:**
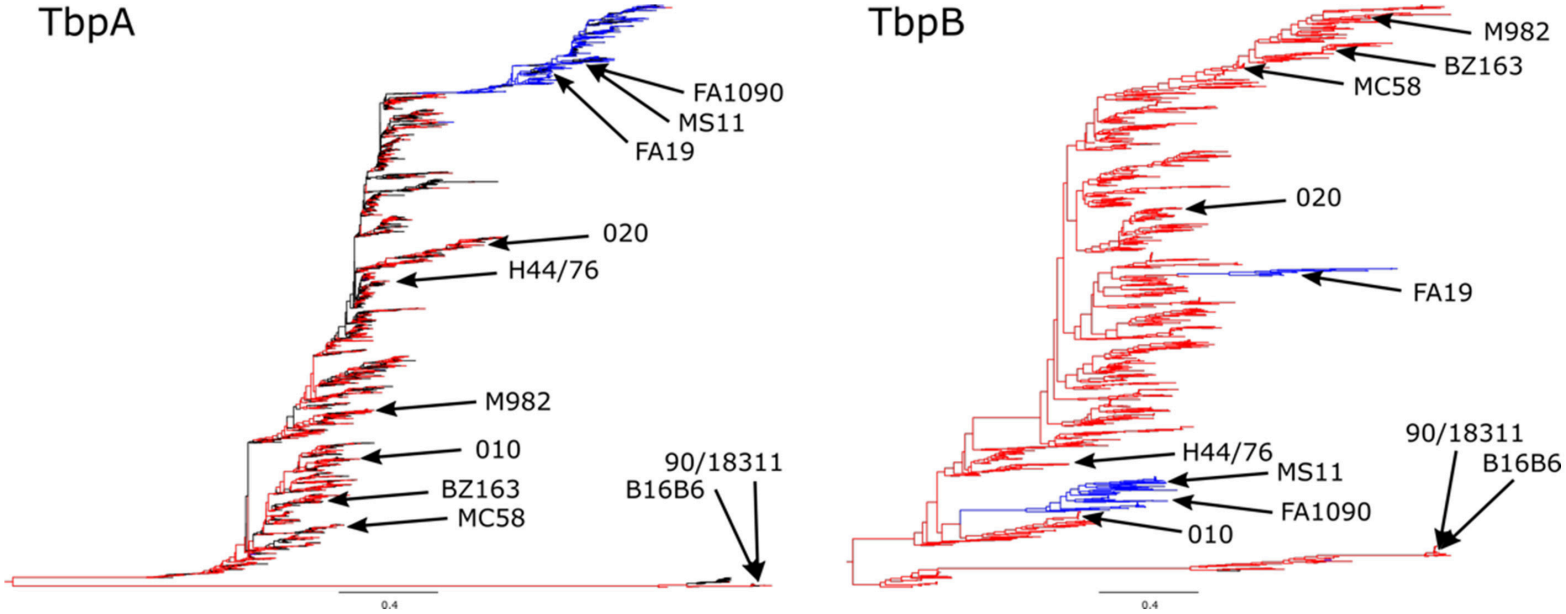
Phylogenetic analysis of Neisseria species TbpA **(Left)** and TbpB **(Right)** protein sequences. *N. meningitidis* sequences are shown in red, *N. gonorrhoeae* sequences are shown in blue, and any commensal *Nesseria* sequences or sequences lacking species annotation are shown in black, with all of the variants described in the (Figure 4) alignment denoted on the trees. The trees are shown at the same scale (0.4) in order to better compare the extent of diversity between the proteins. *N. meningitidis* isotype I TbpBs (including known isotype I containing strains B16B6 and 90/18311) are shown at the bottom with the corresponding TbpAs also shown at the bottom of the trees.

Phylogenetic analysis of TbpA and TbpB sequences from *N. meningitidis* and *N. gonorrhoeae* have demonstrated that the gonococcal diversity exists predominantly as either one subset (TbpA) or two subsets (TbpB) of the isotype II-type meningococcal diversity (Figure 3). When compared at the same scale, TbpB contains substantially more variability than does TbpA within each isotype, however the isotype distinction appears in both TbpA and TbpB sequences, with strains appearing to be consistently isotype I or isotype II, as previously reported (40).

**Figure 4:**
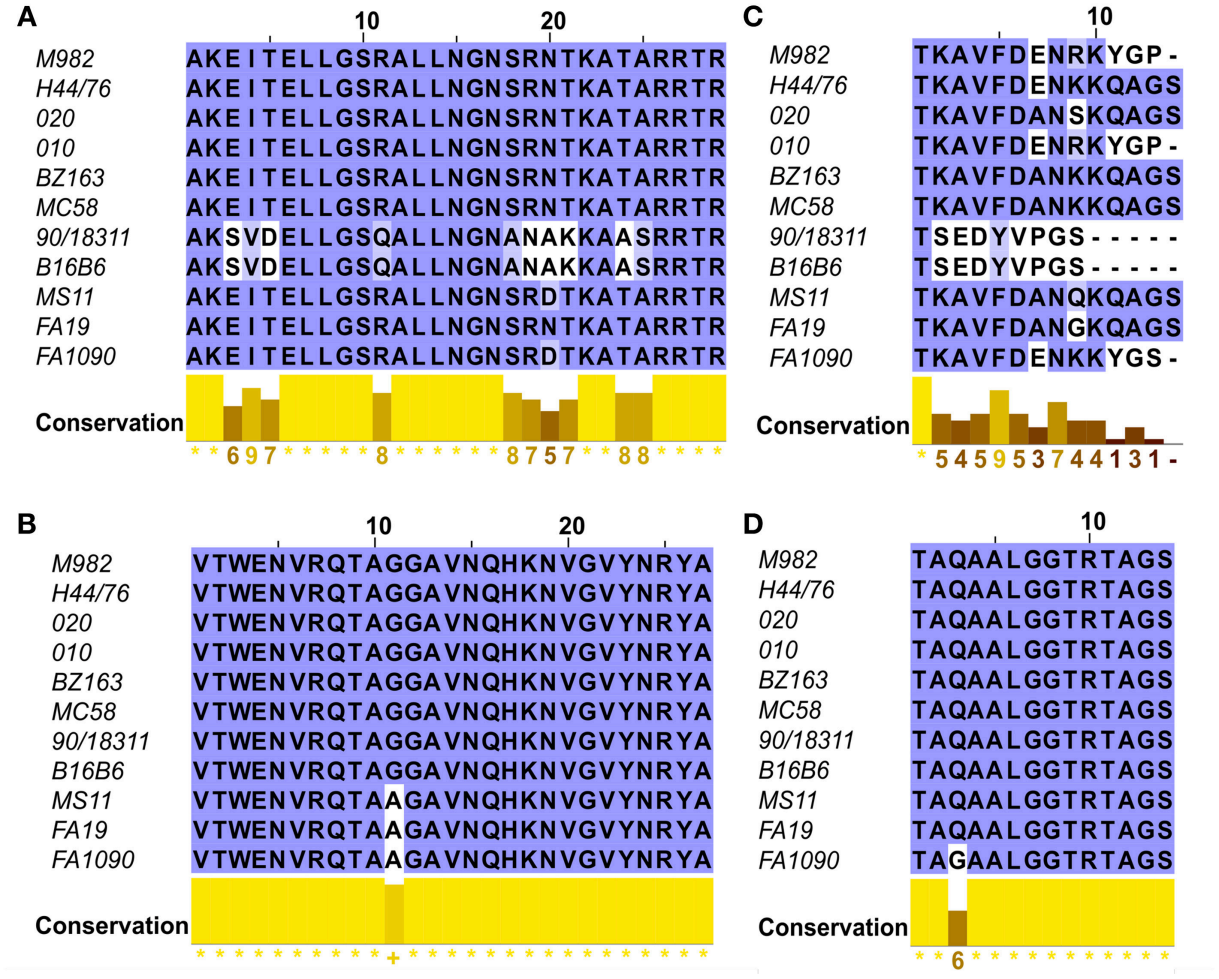
Sequence alignments of the loops of TbpA used in the hybrid antigens across 11 strains including six *N. meningitidis* isotype 2 strains (M982, H44/76, 020, 010, BZ163 and MC58), two *N. meningitidis* isotype 1 strains (B16B6 and 90/18311) and three *N. gonorrhoeae* strains (MS11, FA19 and FA1090). Clustal Omega amino acid alignments displayed in JalView of the four regions of TbpA of interest include **(A)** Loop 10; **(B)** Loop 11; **(C)** Helix 3 and **(D)** the Plug Domain.

To evaluate the potential of hybrid antigens to induce a cross-reactive antibody response, we examined the sequence diversity of the selected loops. The sequences of the chosen TbpA loops were compared across a collection of three *N. gonorrhoeae* strains (Figure 4, bottom three sequences) and eight *N. meningitidis* strains (Figure 4, top eight sequences) selected to represent the overall diversity of *Neisseria* TbpAs (as seen in Figure 3). The overall sequence diversity of TbpA parallels the sequence diversity of the TbpB C-lobe, which divides the C-lobe variants into an isotype I cluster with a smaller size C-lobe (also reflected in the overall TbpB size) and a larger size isotype II C-lobe (40). Thus, the *N. meningitidis* collection included six strains expressing isotype II TbpBs and two strains expressing isotype I TbpBs (B16B6 and 90/18311) while all three *N. gonorrhoeae* strains expressed isotype II TbpBs. There were two variants within loop 11 (Figure 4B) and three variants of loop 10 (Figure 4A), illustrating that these are two of the most conserved surface loops on TbpA. The loop 3 helix region (10 residues) and the plug domain (15 residues) were selected due to the critical function they are proposed to serve in removing iron from Tf (26). In the TbpA-hTf complex, the loop 3 helix is inserted between the C1 and C2 subdomains of transferrin while the plug loop interacts directly with the C1 subdomain, together coordinating the release of iron (26). The plug domain is highly conserved among the representative TbpAs, with only *N. gonorrhoeae* strain FA1090 differing by a single residue (Figure 4D). There is more variability present in the loop 3 helix (Figure 4C), with substantive differences between variants from meningococcal strains expressing isotype I and II TbpBs as well as additional variability within the *N. gonorrhoeae* variants. Each of these four components was spliced into the LCL backbone and each hybrid antigen was expressed, purified (Figure 1C) and used for initial immunizations in mice and rabbits to collect sera for *in vitro* evaluation.

### Ability of Hybrid Antigens to Elicit Functional Antibodies

**Figure 5:**
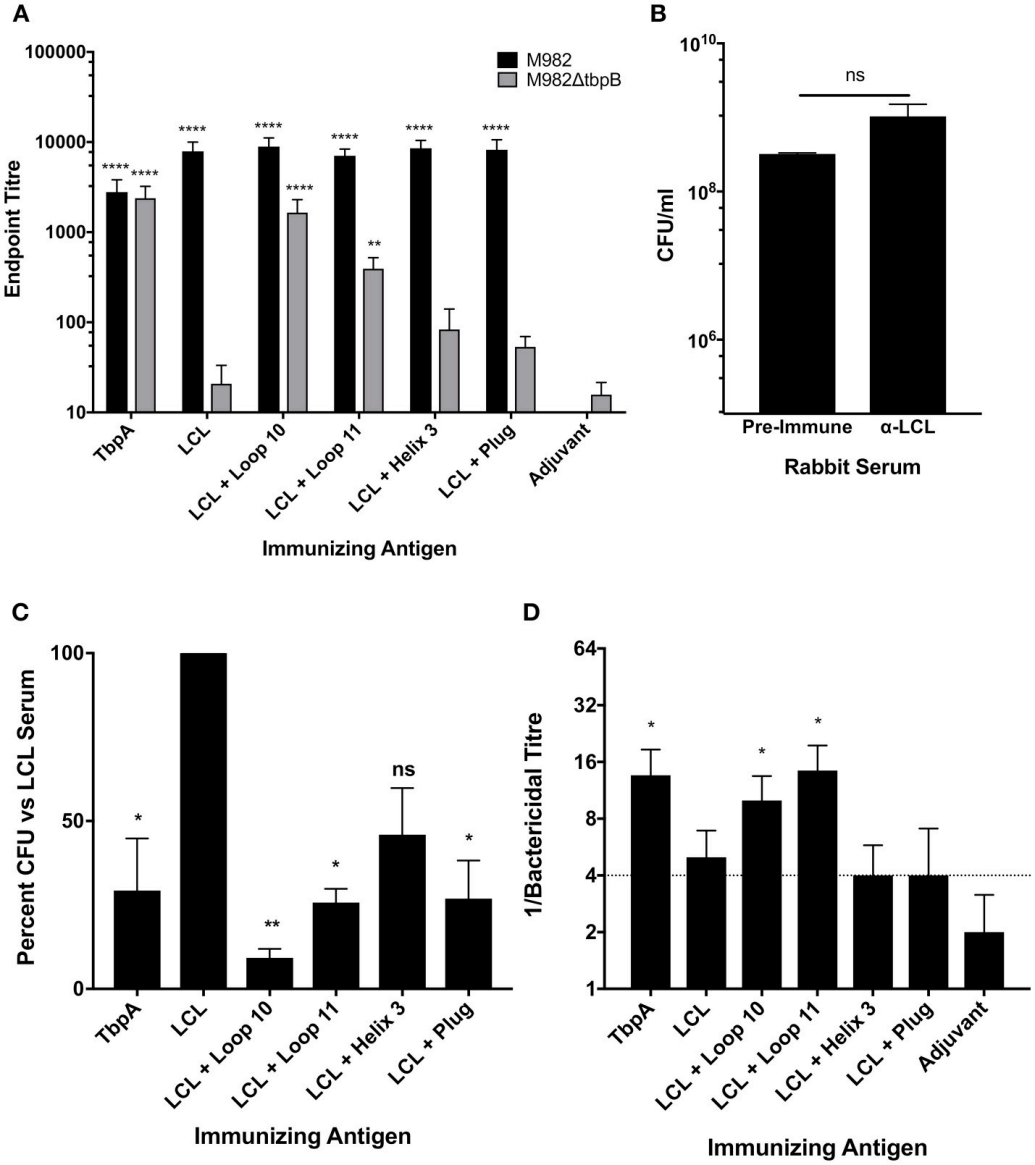
*In vitro* characterization of the LCL scaffold and the LCL-TbpA hybrid antigens. **(A)** Mouse sera were evaluated in a whole cell ELISA against both wild-type *N. meningitidis* M982 (Black) and M982 with TbpB knocked out (M982ΔtbpB; Gray). Sera were assayed in triplicate from individual mice (4 to 5 mice per group) and averaged and displayed as mean +/- SEM. Significance was determined as a significant increase in titer compared with mice that received adjuvant alone by two-way ANOVA with Sidak's multiple comparison test for both wildtype and knockout data sets. **p ≤ 0.01, ****p ≤ 0.0001. **(B,C)** Iron starved *N. meningitidis* M982ΔtbpB was grown with antisera from rabbits immunized with the antigens of interest. Iron-loaded hTf was added and bacteria were grown for 2.5 h and enumerated. Bacteria supplemented with LCL antiserum grew at the same rate as those supplemented with pre-challenge rabbit sera **(B)**, as compared by an unpaired *t*-test. Growth was significantly decreased in bacteria supplemented with whole TbpA antisera, as well as antisera from rabbits immunized with LCL + Loop 10, Loop 11 and the plug domain **(C)**. Graph represents data generated from three independent experiments plated in duplicate. Significance was determined as a significant decrease in final CFU/ml compared with bacteria grown with LCL alone by ordinary one-way ANOVA with *post-hoc* analysis by Holm-Sidak's multiple comparison test. *p ≤ 0.05, **p ≤ 0.01. **(D)** Serum bactericidal activity of mouse sera. Sera from each mouse was assayed against *N. meningitidis* strain M982 lacking TbpB. Data shown are averages of 4 to 5 mice per group ± SEM. A bactericidal titer of ¼ is the limit of detection of bacterial killing. Some values fall below the cut off due to some animals within the group showing killing at the 1:4 dilution while others produced no bacterial killing.

In order to evaluate whether sera raised against the hybrid antigens recognized native epitopes on TbpA at the surface of the bacterial cell, a whole cell ELISA was performed with wild type *N. meningitidis* strain M982 (the homologous strain) and with a TbpB deletion mutant (M982ΔTbpB) to eliminate reactivity due to antibodies against the TbpB-derived scaffold. Sera against the hybrid antigens had higher titres against wild-type M982 (black bars, Figure 5A) than the sera against TbpA, likely reflecting the greater accessibility of TbpB at the cell surface. Notably, anti-loop 10 hybrid titres against M982ΔTbpB (gray bars, Figure 5A) were comparable to the anti-TbpA titres, suggesting that loop 10 is accessible to antibody and that the hybrid antigen efficiently induced antibodies that recognize the native antigen. Antisera raised against loop 11 also displayed significant reactivity against TbpA compared to serum against adjuvant alone. Comparatively low reactivity against M982ΔTbpB was observed with antisera derived from the hybrid loop 3 helix or plug loop immunizations.

To determine if the TbpA loop-specific antibodies raised upon immunization with the hybrid antigens are able to functionally block Tf binding and therefore inhibit growth, the M982ΔTbpB strain was grown with Tf as the sole iron source in the presence of heat-inactivated immune rabbit sera (Figure 5C). We opted to use a strain lacking TbpB to ensure that only antibodies against TbpA were responsible for any observed inhibition of growth. Growth in the presence of antiserum raised against the TbpB-derived LCL was not statistically different from growth in the presence of pre-challenge (naïve) rabbit serum (Figure 5B) and represents baseline, while the 70.7% reduction in CFU with the anti-TbpA antiserum demonstrates that growth inhibition can be achieved. Antisera raised against the loop 11 and plug loop hybrid proteins showed levels of inhibition (74.3 and 73.1%, respectively) similar to that obtained with anti-TbpA; this is particularly notable when considering that the titer of plug-specific antibodies was low, suggesting that antibodies which target it are highly effective at blocking Tf-dependent growth. However, the loop 10 hybrid elicited the strongest growth inhibition, with a reduction in CFU of over 90% compared to bacteria grown in the presence of LCL.

Serum bactericidal assays were performed to quantify complement-dependent killing activity of mouse sera (Figure 5D) against the M982ΔTbpB strain, reflecting bactericidal activity due to anti-TbpA antibodies alone. Using sera from mice immunized with either the loop 10 or loop 11 hybrid proteins, bactericidal activity against the M982ΔTbpB strain occurred in dilutions above 1:8, comparable to the anti-TbpA antisera, indicating that antibodies raised to TbpA loops 10 and 11 alone are sufficient to produce bacterial killing *in vitro*.

### Ability of the Loop 10 Hybrid Antigen to Induce Protection Against Colonization and Sepsis

Since the loop 10 hybrid antigen elicited sera that showed the best reactivity to whole bacterial cells, was bactericidal against TbpA, and was the best at blocking Tf-dependent growth *in vitro*, this antigen was prioritized for evaluation in two separate protection studies. The TbpA loop 10 differs by only one residue between *N. gonorrhoeae* strain MS11 and *N. meningitidis* strain M982 (Figure 4A), suggesting that the same hybrid antigen could be evaluated against both lower genital tract infection by *N. gonorrhoeae* and invasive challenge by *N. meningitidis*. The TbpA loop 10 from *N. meningitidis* strain M982 was transplanted onto a foreign TbpB from the porcine pathogen *Haemophilus parasuis* (Figure 1D) to avoid the immune response against TbpB induced by the LCL obscuring protection mediated by the TbpA loop 10 when challenging with wild type bacteria (expressing *Neisseria* TbpB).

**Figure 6:**
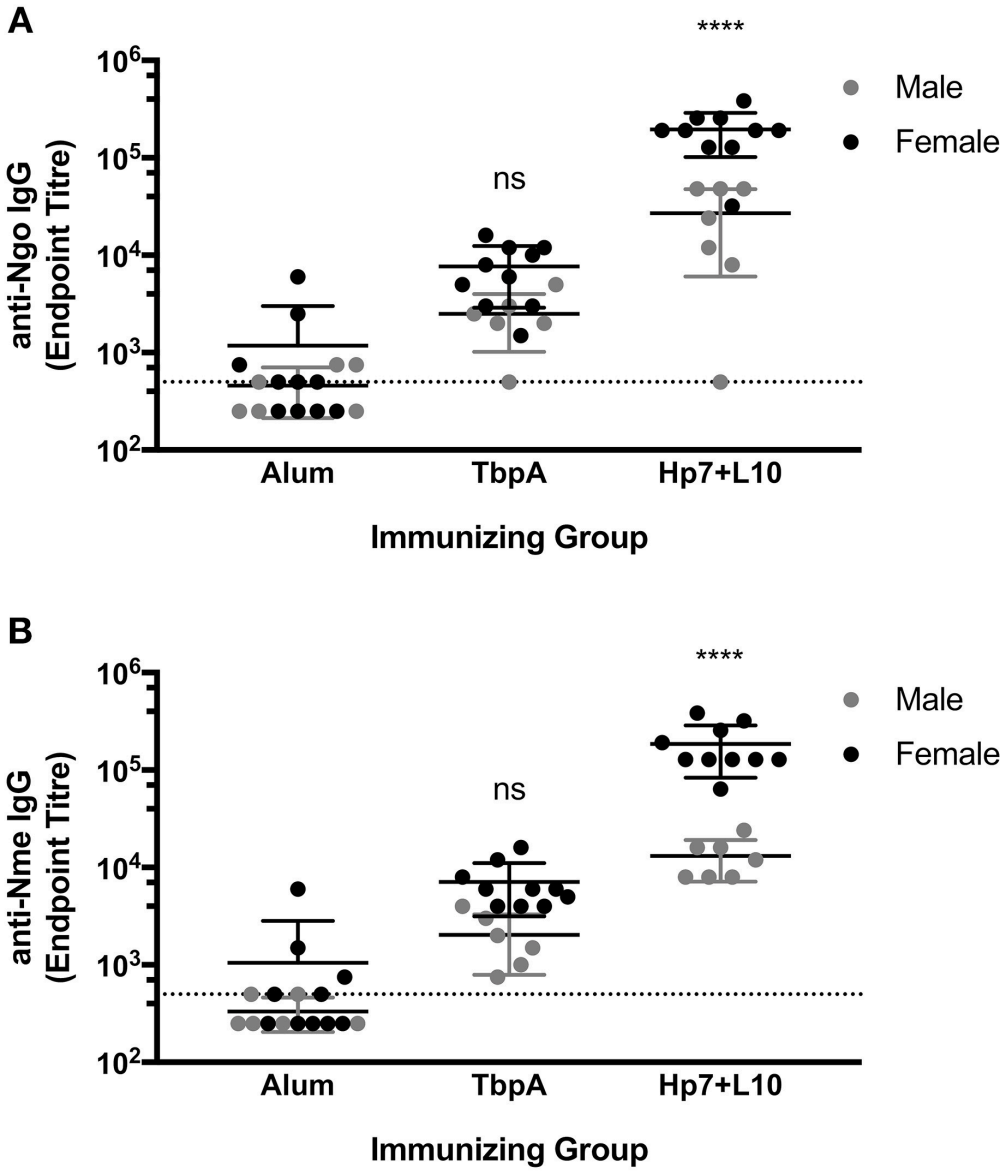
Whole-cell serum IgG ELISA titres of mice immunized with alum, TbpA or Hp7-L10 when captured by **(A)** heat-killed *N. gonorrhoeae* strain MS11 or **(B)** heat-killed *N. meningitidis* strain M982. Female mice were used in the lower genital tract gonococcal colonization, while male mice were challenged systemically with *N. meningitidis*. Significance was determined as a significant increase in titer compared with mice that received alum alone by ordinary one-way ANOVA with *post-hoc* analysis by Dunnett's multiple comparisons test. ****p < 0.0001.

**Figure 7:**
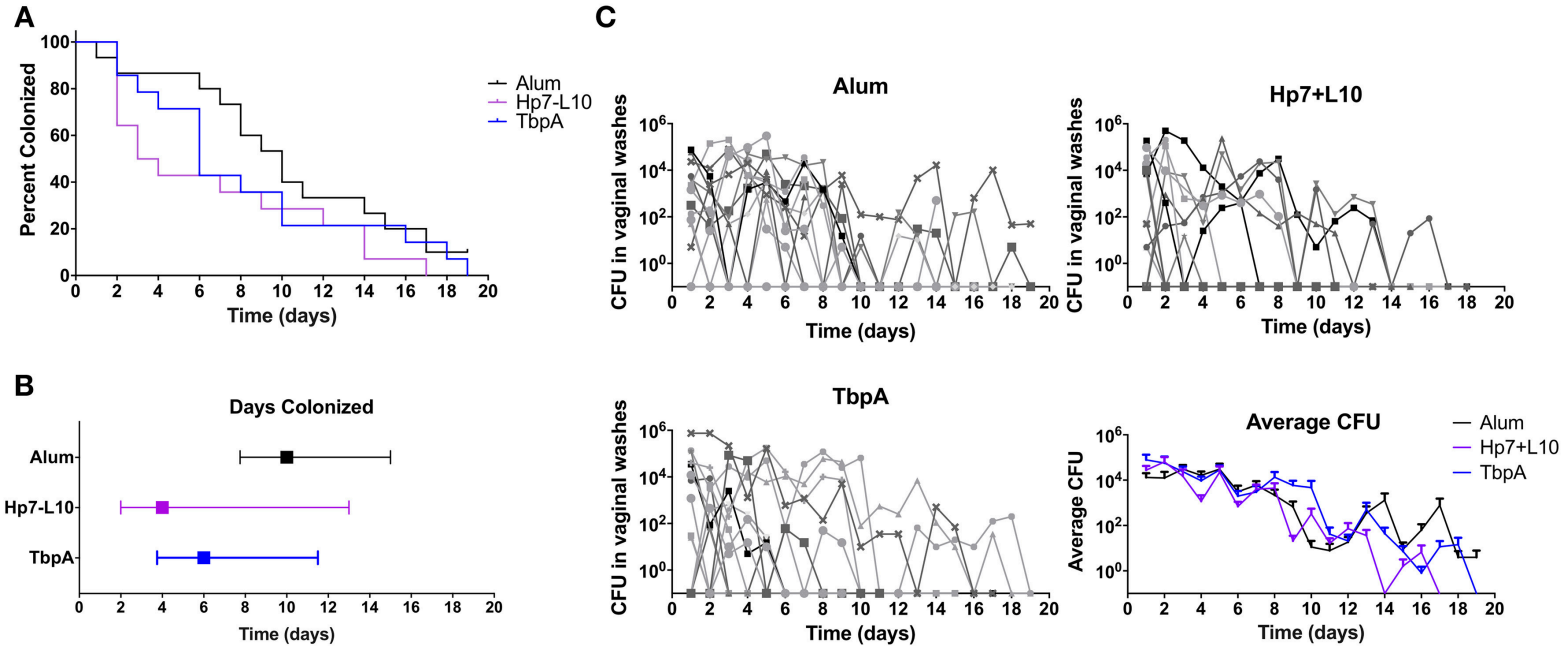
Lower genital tract gonococcal colonization in immunized female mice. Mice were immunized with one of TbpA (17 mice, with 2 excluded due to non-infection; blue), *H. parasuis* TbpB hosting the meningococcal TbpA loop 10 (17 mice, with 5 excluded due to non-infection; purple) or alum alone (14 mice, with 5 excluded due to non-infection; black) and challenged with 10^7^ CFU of *N. gonorrhoeae* intra-vaginally. Mice were immunized and challenged in two cohorts and are shown as combined data. Duration of colonization is shown as percent **(A)** as well as by median with interquartile range **(B)**. The recovered CFU from vaginal washes from each mouse is shown **(C)** broken down by each immunization group as well as averages are shown. Duration of gonococcal colonization was compared by a log rank [Mantel-Cox] test, however no significance was observed.

To test for protection against *N. gonorrhoeae* infection in the female lower genital tract, recombinant TbpA from *N. meningitidis* strain M982 or the *H. parasuis* TbpB hosting the *Neisseria* loop 10 were used to immunize wild type, female C57Bl/6 mice (**Figure 7**). *H. parasuis* TbpB hosting the *Neisseria* loop 10 was highly immunogenic in mice as shown by serum IgG levels against heat-killed *N. gonorrhoeae* (Figure 6A) and *N. meningitidis* (Figure 6B) in a whole-cell ELISA, while mice immunized with TbpA showed an increase in reactivity against whole bacteria that did not reach statistical significance compared to mice immunized with alum alone. The immunized female C57Bl/6 mice were challenged intra-vaginally with 10^7^ CFU of *N. gonorrhoeae* strain MS11. Mice that received alum were robustly colonized with a median colonization period of 10 days between the two cohorts (Figures 7A,B). In contrast, mice immunized with *Neisseria* TbpA had a median colonization duration of 6 days, while mice immunized with the *H. parasuis* TbpB hosting loop 10 had a median colonization duration of only 4 days.

**Figure 8:**
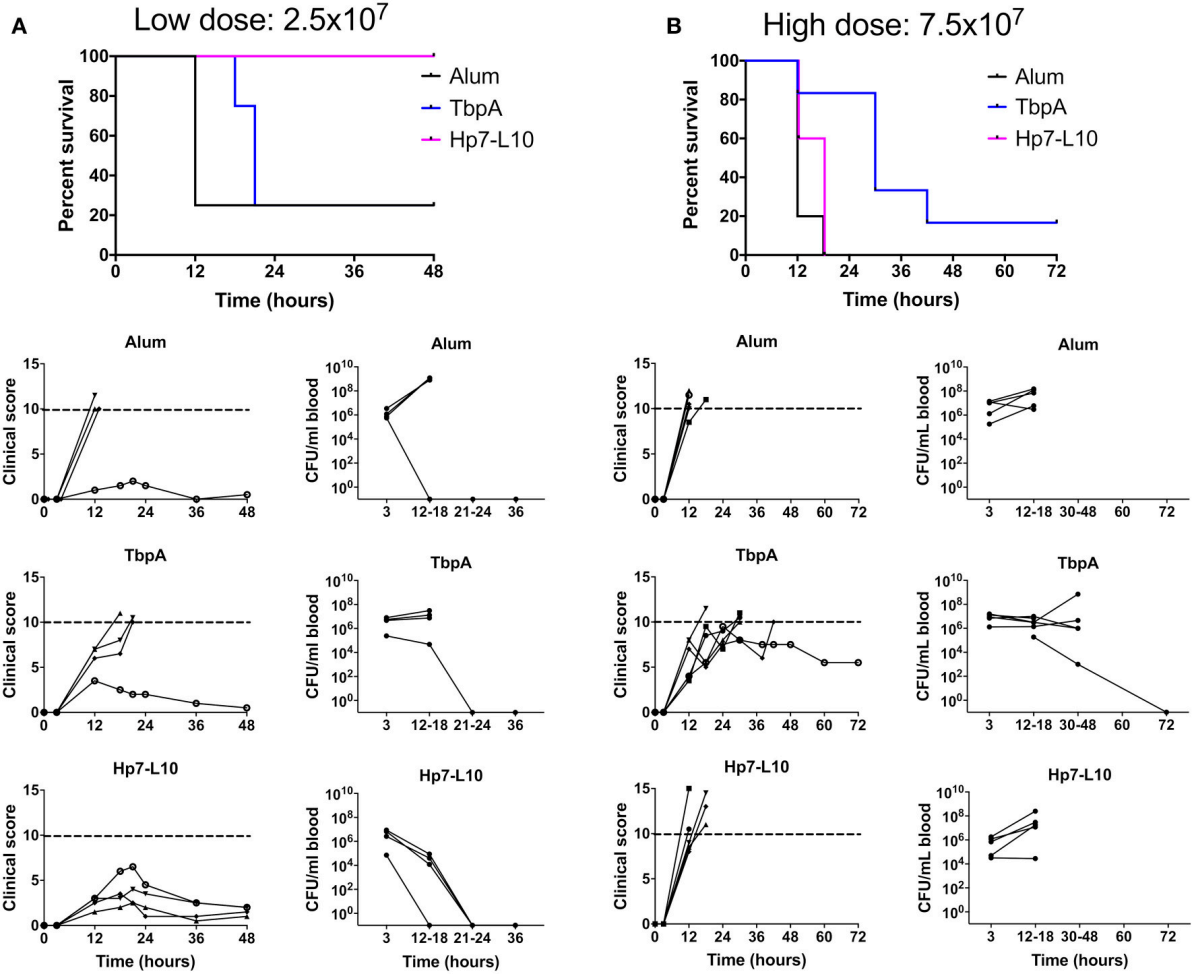
Survival of immunized mice in a model of invasive meningococcal infection of mice immunized with either the low dose [**(A)**; 2.5 × 10^7^ CFU per mouse] or the high dose [**(B)**; 7.5 × 10^7^ CFU/mouse]. Mice were immunized with one of TbpA (blue), *H. parasuis* TbpB hosting the meningococcal TbpA loop 10 (Hp7-L10; purple) or alum alone (black) and challenged with a lethal dose of *N. meningitidis* strain M982 systemically. Mouse survival is shown at the top, with clinical scores and bacterial burden in the blood shown for individual mice for each immunization group (descending from the top): alum, TbpA, and *H. parasuis* TbpB hosting loop 10.

To measure protection against invasive meningococcal disease, male C57Bl/6 mice were immunized with the two antigens or alum, and then challenged parenterally with *N. meningitidis* strain M982, the homologous strain to the antigens in use (Figure 8). In this experiment mice were infected in two cohorts with either a low (2.5 × 10^7^ CFU per mouse) or high (7.5 × 10^7^ CFU per mouse) dose of *N. meningitidis*. The hybrid antigen displaying loop 10 conferred protection superior to that with TbpA upon low dose challenge; all mice immunized with the hybrid antigen survived the low dose challenge, had relatively low clinical scores, and there were no bacteria detected in blood after 24 h (Figure 8A). In contrast, the mice immunized with this hybrid antigen challenged at the high dose had a similar outcome to the adjuvant treated mice (Figure 8B). In future experiments, it will be important to determine whether using a combination of hybrid antigens displaying different loops at different sites on TbpB can overcome the lack of protection observed when the immune response is directed against a single hybrid antigen.

The results with mice immunized with the native TbpA was surprising in that there was little protection observed at the low challenge dose (Panel A) and a modest level of protection observed at the high challenge dose (Panel B). Since the TbpA used in the low dose and high dose experiments were from different preparations, we cannot exclude the possibility that the low and variable level of protection observed could be due to differences in the conformation and state of the protein used for immunization or, alternatively, due to formulation effects such as its association with the adjuvant. It is noteworthy that early experiments suggested that TbpA would not be protective (11, 15), likely due to inconsistencies in the conformation and stability of the protein. Subsequent studies demonstrated effective protection, and heterologous strain cross-protection, against challenge in the sepsis model (22). Collectively, these results highlight the challenges that could be encountered for commercial production of a TbpA antigen.

## Discussion

The pursuit of a vaccine for prevention of gonorrhea has been a long-standing goal, with early efforts at developing a pilus-based vaccine culminating in disappointing results in a clinical trial (41). In spite of some pessimism at the time regarding the feasibility of developing a gonococcal vaccine, continued efforts by multiple groups have led to a number of promising targets in the intervening years (42). The growing prevalence of strains resistant to multiple antibiotics is increasing the potential for non-treatable cases of gonorrhea (43), providing a renewed sense of urgency for the development of an effective vaccine for disease prevention.

The development and implementation of an experimental gonococcal infection model in male volunteers (44) has been an invaluable tool for providing insights into the interaction between gonococci and the host, and in evaluating potential vaccine targets. The ability of gonococcal strains deficient in IgA protease, pili (PilE), or Opa proteins to remain infectious in this model suggests that these proteins are not essential and thus may not be ideal vaccine targets, although one cannot exclude the possibility that they mediate critical steps in the natural infectious process. In contrast, a strain deficient in the transferrin receptor was unable to persist (1), which at the time was unexpected since the concept of transferrin being inside the body and lactoferrin being present on the mucosal surface was the prevailing view of iron homeostasis. Since this experiment was performed with a gonococcal strain naturally lacking a functional lactoferrin receptor, a follow up study was initiated to compare the roles of these two iron acquisition systems (2). Restoration of a functional lactoferrin receptor in the strain lacking the transferrin receptor enabled it to establish infection in a substantial proportion of the volunteers, demonstrating that either receptor could support growth on the mucosal surface of the male genitourinary tract.

A competition study with strains expressing a functional Tf receptor and either possessing or lacking a functional Lf receptor demonstrated that all of the persisting bacteria had both receptors (2). Although it was not possible to establish a mechanism for this competitive advantage, Lf binding protein B (LbpB)-mediated protection against cationic peptides may have contributed to this advantage (39). Indirect assessment of mucosal concentrations of Tf and Lf by measuring their concentrations in urine indicated that, prior to infectious challenge, the median concentration of Tf was 2.1-fold higher than Lf (2). This indicates that Tf is present on the mucosal surface in the absence of significant inflammation, however the mechanism for delivery of Tf to the mucosal surface remains unknown. Several days after challenge, the levels of Lf, but not of Tf, rose. The rise in Lf is consistent with its release by degranulating neutrophils at the site of infection, and the lack of increase of Tf suggests that the delivery of Tf to the mucosal surface is not due to leakage during inflammation.

Although we do not have any information regarding the specific role of each component of the Tf receptor from the human male urethral infection model, studies with the pig pathogen *Actinobacillus pleuropneumoniae* indicate that TbpB is essential for persistence and infection in the porcine respiratory tract (45). The invariant presence of TbpB in *N. gonorrhoeae, N. meningitidis* and all other human and animal pathogens that possess the bipartite TbpB-TbpA receptor studied to date make clear that TbpB is essential for the efficient capture of iron-containing Tf on the mucosal surface. Recent studies with a second pig pathogen, *H. parasuis*, demonstrated that a Tf binding-defective mutant of TbpB elicited dramatically superior protection relative to either native TbpB or a commercially-available vaccine in an invasive infection model (20). These results provide an explanation for the disappointing results from a Phase I trial in humans (17), which used a binding-competent TbpB from *N. meningitidis*; a binding-defective mutant would presumably also be superior in humans. Through the bioinformatic analysis of TbpB diversity from three porcine pathogens, immunologic analysis of the cross-reactivity of the humoral response, and additional immunization and challenge experiments (21, 46), the prospect of developing a porcine TbpB-based vaccine with two or three mutant TbpBs for prevention of infection by three pathogens seems feasible. In addition, recent preliminary results suggest that immunization of piglets in a production facility with TbpB-based vaccine formulations can result in eliminating natural colonization. Such sterilizing immunity is a prerequisite for any gonococcal vaccine since a vaccine that elicits asymptomatic carriage would only facilitate transmission of this already pervasive pathogen.

Analysis of the diversity of TbpB from strains of *N. gonorrhoeae* and other *Neisseria* species (Figure 3) suggests that a TbpB-based vaccine derived from two or three representative TbpBs could induce a protective response against all gonococcal isolates. However, since the gonococcus can be isolated from the upper respiratory tract and strains of *N. meningitidis* have been isolated from cases of urethritis (47), there may be ample opportunity for horizontal genetic exchange in response to strong selective pressures. Thus, the long-term efficacy of a Tf receptor-based vaccine may ultimately need to consider the reservoir of *tbpA* and *tbpB* genes in other *Neisseria* species (Figure 3), requiring a larger number of representative variants for a pan-*Neisseria* vaccine.

The more limited variation in TbpA sequences and its critical role in the essential iron acquisition process make it an attractive target for a gonococcal vaccine. Our *N. gonorrhoeae* colonization studies in a female mouse model showed a reduction in colonization due to immunization with TbpA (Figure 7) or a foreign TbpB displaying the *Neisseria* TbpA loop 10, suggesting that our hybrid antigen strategy has potential to elicit protection at the mucosal surface. It is noteworthy to mention that the *N. gonorrhoeae* challenge strain in the mouse model used in this study is not dependent upon its Tf receptor for growth in this model since only mouse (and not human) Tf is present. Thus, based on what has been observed in human studies (1), in which sustained growth and infection requires acquisition of iron from host proteins, ongoing bacterial replication in the mouse model is likely dependent upon the substantial iron stores from growth of the large inoculums on iron rich media prior to challenge. This clearly contrasts the more physiologically-relevant situation in the human male urethral model for *N. gonorrhoeae* or in the upper respiratory tract of pigs for *H. parasuis*, where bacterial survival and replication is Tf-dependent; in these cases, the anti-Tf receptor antibodies might also play a role in inhibiting the growth of the bacteria. Therefore, subsequent studies are needed utilizing transgenic mice expressing human Tf in place of mouse Tf, where the inoculum may contain bacteria with depleted levels of stored iron (achieved via growth of the bacteria in the presence of an iron chelator prior to challenge). In this instance, ongoing infection will require iron acquisition, which should reveal an even greater impact of Tbp-specific antibodies.

One other aspect that remains to be assessed in future studies is the effect of varying adjuvants. The use of aluminum hydroxide as the vaccine adjuvant in these challenge studies (chosen since it is licensed for humans) may not be optimal for inducing a protective response, and other adjuvants may provide more rapid bacterial clearance. Indeed, recent work by Russell and colleagues demonstrates that cytokine-induced polarization of the immune response can facilitate protection against *N. gonorrhoeae* infection (48, 49). Novel mucosal adjuvants are being developed in order to better target immune responses to the site of infection (50), which may be able to elicit a more robust anti-gonococcal response. Additionally, aluminum hydroxide is known to skew the immune response in a T_H_2 dependent manner (51) that results in high levels of IgG1 and limited production of IgG isotypes such as mouse IgG2a or IgG2c. IgG1 has been shown to inhibit the binding of other mouse IgG isotypes to the complement factor C1q, the molecule that initiates the classical complement pathway, and thus limits the induction of IgG2a-mediated complement activation (52). This may explain why the loop 10 hybrid antigen was protective against a low but not a high dose of *N. meningitidis* in the invasive challenge model (Figure 8), where at a higher dose a partial inhibition of complement activation was sufficient to limit protection.

The ability of a soluble TbpB-based scaffold antigen to support exogenous loops from integral membrane proteins provides the potential to broaden the prospective breadth of antigens that can be targeted with a subunit vaccine. As the Lf receptor was able to rescue gonococcal survival in the human male urethra in the absence of the Tf receptor, expanding the scope of these antigens to contain surface exposed loops of Lf binding protein A (LbpA) may provide even broader protection while limiting the potential for vaccine escape by gonococcal isolates that express the Lf receptor.

This study demonstrated that an engineered TbpB antigen is able to display epitopes from TbpA and provide stable, immunogenic antigens that can produce anti-TbpA antibodies that are bactericidal and able to inhibit Tf-dependent growth. The proof of concept provided by this study suggests that a more comprehensive analysis of different surface loops, and of combinations of loops, will allow for an optimized immune response against TbpA. It will be important to independently assess whether the display of TbpA epitopes interferes with the cross-reactive anti-TbpB response in order to make sure the cross-protective properties of the mutant TbpBs are not compromised by the display of heterologous loops; this could presumably be overcome by including a mixture of two or more antigens with insertions at different sites. In summary, this strategy establishes the utility of our novel method of increasing the breadth of vaccine targets against diverse naturally occurring receptor variants by specifically targeting areas of interest on membrane proteins and provides the exciting potential for a single Tbp-based vaccine protective against both meningococcal and gonococcal infection.

## Author Contributions

AS conceived the original idea and oversaw all aspects of the study. AS consulted with TM, CC, and SC on the design of the hybrid antigens, oversaw the preparation and production of hybrid antigens by RY, and provided advice and guidance to JF in the immunization and evaluation of the immune response. TM provided oversight in the design of hybrid antigens by CC. CC examined the structures and designed the epitope transfers with input from AS and TM. RY and SC prepared the hybrid antigen genes, expressed and purified the recombinant hybrid antigens. JF performed all the functional studies with the sera including recognition of TbpA in intact cells, inhibition of growth and bactericidal activity. JF, EI, and SA designed and performed the mouse protection studies under the guidance of SG-O. JF prepared the initial draft of the manuscript and JF and AS finalized the manuscript. JF and EI prepared all figures in the manuscript.

### Conflict of Interest Statement

AS, SG-O, and TM are inventors on a patent that described the design and production of the hybrid antigen and co-owners of Engineered Antigens Inc. The remaining authors declare that the research was conducted in the absence of any commercial or financial relationships that could be construed as a potential conflict of interest.
